# Plasma microRNA Environment Linked to Tissue Factor Pathway and Cancer-Associated Thrombosis: Prognostic Significance in Ovarian Cancer

**DOI:** 10.3390/biom14080928

**Published:** 2024-07-31

**Authors:** Valéria Tavares, Joana Savva-Bordalo, Mariana Rei, Joana Liz-Pimenta, Joana Assis, Deolinda Pereira, Rui Medeiros

**Affiliations:** 1 Molecular Oncology and Viral Pathology Group, Research Center of IPO Porto (CI-IPOP)/Pathology and Laboratory Medicine Dep., Clinical Pathology SV/RISE@CI-IPOP (Health Research Network), Portuguese Oncology Institute of Porto (IPO Porto)/Porto Comprehensive Cancer Centre (Porto. CCC), 4200-072 Porto, Portugal; valeria.tavares@ipoporto.min-saude.pt; 2 ICBAS—Instituto de Ciências Biomédicas Abel Salazar, Universidade do Porto, 4050-313 Porto, Portugal; 3 Faculty of Medicine, University of Porto (FMUP), 4200-072 Porto, Portugal; jpimenta@chtmad.min-saude.pt; 4 Department of Medical Oncology, Portuguese Institute of Oncology of Porto (IPO Porto), 4200-072 Porto, Portugal; joana.sa@ipoporto.min-saude.pt (J.S.-B.); dpereira@ipoporto.min-saude.pt (D.P.); 5 Department of Gynaecology, Portuguese Institute of Oncology of Porto (IPO Porto), 4200-072 Porto, Portugal; marianacruzrei@gmail.com; 6 Department of Medical Oncology, Centro Hospitalar de Trás-os-Montes e Alto Douro (CHTMAD), 5000-508 Vila Real, Portugal; 7 Clinical Research Unit, Research Center of IPO Porto (CI-IPOP)/RISE@CI-IPOP (Health Research Network), Portuguese Oncology Institute of Porto (IPO Porto)/Porto Comprehensive Cancer Center (Porto. CCC), 4200-072 Porto, Portugal; joana.assis@ipoporto.min-saude.pt; 8 Faculty of Health Sciences, Fernando Pessoa University, 4200-150 Porto, Portugal; 9 Research Department, Portuguese League Against Cancer (NRNorte), 4200-172 Porto, Portugal

**Keywords:** ovarian neoplasms, thrombosis, prognosis, microRNAs, liquid biopsy

## Abstract

Ovarian cancer (OC) is a leading cause of death among gynaecological malignancies. The haemostatic system, which controls blood flow and prevents clotting disorders, paradoxically drives OC progression while increasing the risk of venous thromboembolism (VTE). MicroRNAs (miRNAs) have emerged as crucial in understanding VTE pathogenesis. Exploring the connection between cancer and thrombosis through these RNAs could lead to novel biomarkers of cancer-associated thrombosis (CAT) and OC, as well as potential therapeutic targets for tumour management. Thus, this study examined the impact of eight plasma miRNAs targeting the tissue factor (TF) coagulation pathway—miR-18a-5p, -19a-3p, -20a-5p, -23a-3p, -27a-3p, -103a-3p, -126-5p and -616-3p—in 55 OC patients. Briefly, VTE occurrence post-OC diagnosis was linked to shorter disease progression time (log-rank test, *p* = 0.024) and poorer overall survival (OS) (log-rank test, *p* < 0.001). High pre-chemotherapy levels of miR-20a-5p (targeting *coagulation factor 3* (*F3*) and *tissue factor pathway inhibitor 2* (*TFPI2*)) and miR-616-3p (targeting *TFPI2*) predicted VTE after OC diagnosis (χ^2^, *p* < 0.05). Regarding patients’ prognosis regardless of VTE, miR-20a-5p independently predicted OC progression (adjusted hazard ratio (aHR) = 6.13, *p* = 0.005), while miR-616-3p significantly impacted patients’ survival (aHR = 3.72, *p* = 0.020). Further investigation is warranted for their translation into clinical practice.

## 1. Introduction

In 2022, there were nearly 324,000 newly diagnosed cases of ovarian cancer (OC) worldwide, with approximately 206,000 related deaths. This malignancy stands as the eighth deadliest cancer among women, being the most lethal gynaecological tumour [1,2]. Furthermore, the prognosis of OC patients is worsened by the occurrence of cancer-associated thrombosis (CAT), particularly venous thromboembolism (VTE) [3,4].

In the tumour microenvironment, cancer cells interact with key haemostatic players, including endothelial cells, platelets and plasma proteins. In physiological conditions, these components prevent haemorrhage and thrombosis while ensuring endothelium integrity [5]. However, their complex interplay with cancer cells and leukocytes can disrupt the haemostatic balance, augmenting the thrombogenic and bleeding potential. In parallel, haemostatic abnormalities can fuel tumour neo-angiogenesis, cancer-promoted inflammation, immune escape and metastasis [5,6]. Indeed, a two-way association between cancer and VTE, known as the Trousseau syndrome, is well-characterised [4]. Regarding OC patients, they have an incidence of VTE events, primarily deep venous thrombosis (DVT) and pulmonary embolism (PE), of approximately 20%, placing them among those most affected by CAT [3].

MicroRNAs (miRNAs) are single-stranded and highly conserved non-coding RNAs with 18 to 25 nucleotides that control gene expression at a post-transcriptional level. They primarily bind to the 3′ UTR of target messenger RNAs (mRNAs), leading to translation inhibition and potential mRNA degradation [5]. A single miRNA can target the expression of several genes, with a wide range of biological implications. Conversely, a single gene can be the target of multiple miRNAs [7,8]. Given their roles and characteristics, miRNAs have emerged as attractive disease biomarkers. Their high sensitivity and elevated stability in biofluids, even with pH and temperature variations, freeze-thaw cycles and RNase treatment, make them particularly attractive for liquid biopsy applications [5]. In addition, their ability to target several biological pathways puts them at the forefront of non-coding RNA-based therapeutics [7].

Like other biological processes, haemostasis is controlled by miRNAs, which regulate the expression of plasma proteins and the activity of platelets, endothelial cells and leucocytes. Consistently, serum/plasma miRNAs have been implicated in VTE pathogenesis in both the general population and cancer patients. Several of them target essential members of the tissue factor (TF) pathway (also known as the extrinsic coagulation pathway): TF, tissue factor pathway inhibitor 1 (TFPI1 or simply TFPI) and tissue factor pathway inhibitor 2 (TFPI2) [5]. Overexpression of TF (coagulation factor III) is documented in various solid tumours, including OC, promoting both venous thrombogenesis and ovarian tumourigenesis [9,10,11,12,13,14]. The anticoagulant TFPI1 is the main inhibitor of the TF pathway, while TFPI2 can additionally act as a plasmin inhibitor, regulating fibrinolysis and the remodelling of endothelial cell matrix (ECM) [5]. TFPI1 and TFPI2 are deemed tumour suppressors, yet their role can vary depending on the cancer type and its specific molecular environment [5,15,16]. Considering the involvement of miRNAs in the Trousseau syndrome, this study explored the impact of peripheral blood-based miRNAs (i.e., circulating miRNAs) on CAT occurrence and patients’ prognosis (regardless of VTE).

## 2. Materials and Methods

### 2.1. Patients

This retrospective cohort study is part of a broader research protocol exploring CAT biomarkers and their implications in OC patients [17,18]. The study included adult Caucasian patients with confirmed epithelial ovarian carcinoma (EOC) admitted at the Clinic of Gynaecology of the Portuguese Oncology Institute of Porto (IPO Porto) between March 2017 and September 2023 for initial treatment. The treatment protocol included cytoreductive surgery and platinum-based chemotherapy. The number of cycles varied based on the treatment approach (neoadjuvant, adjuvant or as a standalone therapy for patients who were ineligible for surgery). Exclusion criteria encompassed: (1) a history of previous and concurrent tumours, (2) immunosuppression and/or autoimmune disorders, (3) acute infections upon OC diagnosis, (4) pregnancy or postpartum status (up to six weeks after childbirth) at diagnosis, (5) anticoagulation therapy unrelated to VTE, (6) presence of relevant VTE-related genetic polymorphisms, specifically prothrombin G20210A or factor V Leiden and (7) refusal to provide informed consent. A total of 55 OC patients, whose biological samples were stored in the institutional biobank, were included in the study. Their demographical and clinical factors were revised using electronic medical records (Table 1). Each patient signed a written informed consent following the principles of the Helsinki Declaration. The ethics committee at the research centre of IPO Porto (CI-IPO Porto) approved the study (CES. 69/021).

CAT was defined as an event occurring within the period of six months before to two years after the cancer diagnosis [18]. Active screening for OC-related VTE was not performed as it is not part of the standard clinical procedures at IPO Porto. The median follow-up time was 27.0 months (minimum = 1.0 months; maximum = 85.0 months).

### 2.2. miRNA Selection

A comprehensive review of all miRNAs associated with VTE in the general population and among cancer patients was conducted to identify the most suitable miRNAs to be evaluated [5]. From the initial list, only those targeting *coagulation factor 3* (*F3*), *TFPI1* and *TFPI2* were considered, given their pivotal role in CAT. Furthermore, miRNAs evaluated in plasma samples and with more evidence (i.e., reported in several studies) were prioritised. Data from studies on other pathophysiological conditions were gathered from the literature to explore and confirm the specific miRNA–mRNA interactions. Additionally, several miRNA databases were utilised, including the latest versions of TargetScanHuman (v8), miRDB (v6), miRmap (v1.1), miRWalk (v3), DIANA-TarBase (v9) and miRTarBase (v9.0), with all resources last accessed on 6 March 2024. Based on these criteria, hsa-miR-18a-5p, hsa-miR-19a-3p, hsa-miR-20a-5p, hsa-miR-23a-3p, hsa-miR-27a-3p, hsa-miR-103a-3p, hsa-miR-126-5p and hsa-miR-616-3p were selected (Table 2).

### 2.3. Blood Sample Collection and Processing

Peripheral blood samples were obtained from each patient before initiating the first-line chemotherapy (baseline) using a standard venipuncture method. The collected samples, stored in EDTA-coated tubes, underwent centrifugation at room temperature for 5 min at 3000 rpm to isolate the plasma fraction. The prepared plasma samples were kept at −80 °C until needed.

### 2.4. Total RNA Extraction and cDNA Synthesis

Total RNA was extracted from plasma samples using the MagMAX™ mirVana™ Total RNA Isolation Kit (CAT A27828, Thermo Fisher Scientific, Waltham, MA, USA) in a KingFisher™ Duo Prime Magnetic Particle Processor (Thermo Fisher Scientific, Waltham, MA, USA). RNA purity and concentration were evaluated using the NanoDrop Lite spectrophotometer (Thermo Fisher Scientific, Waltham, MA, USA). Following extraction, RNA samples were stored at −80 °C until use.

RNA samples (50 ng) were reversely transcribed into complementary DNA (cDNA) using the TaqMan™ MicroRNA Reverse Transcription Kit (CAT 4366596, Thermo Fisher Scientific, Carlsbad, CA, USA) and the sequence-specific stem-loop reverse transcription primers for hsa-miR-18a-5p (assay ID 002422), hsa-miR-19a-3p (assay ID 000395), hsa-miR-20a-5p (assay ID 000580), hsa-miR-23a-3p (assay ID 000399), hsa-miR-27a-3p (assay ID 000408), hsa-miR-103a-3p (assay ID 000439), hsa-miR-126-5p (assay ID 000451), hsa-miR-616-3p (assay ID 002414), U6 snRNA (assay ID 001973), hsa-miR-1228-3p (assay ID 002919) and hsa-miR-451a (assay ID 001141), following the manufacturer’s instructions. The conversion was performed in a Mycycler^TM^ Thermal cycler (Bio-Rad Laboratories, Hercules, CA, USA) with the following cycle conditions: 30 min at 16 °C, 60 min at 42 °C and 10 min at 85 °C. To evaluate potential contaminations, controls devoid of RNA were included in all conversion reactions.

### 2.5. Relative Quantification of miRNAs

The miRNA expression was quantified via real-time polymerase chain reaction (RT-PCR) in a StepOnePlus^TM^ qPCR system (Applied Biosystems^®^, Foster City, CA, USA). Each PCR reaction was conducted using 5.0 µL of 2× TaqMan™ Fast Advanced Master Mix (Applied Biosystems^®^, Foster City, CA, USA), 2.5 µL of nuclease-free water, 0.5 µL of 20× specific TaqMan™ MicroRNA Assays for hsa-miR-18a-5p (assay ID 002422), hsa-miR-19a-3p (assay ID 000395), hsa-miR-20a-5p (assay ID 000580), hsa-miR-23a-3p (assay ID 000399), hsa-miR-27a-3p (assay ID 000408), hsa-miR-103a-3p (assay ID 000439), hsa-miR-126-5p (assay ID 000451), hsa-miR-616-3p (assay ID 002414), U6 snRNA (assay ID 001973), hsa-miR-1228-3p (assay ID 002919) and hsa-miR-451a (assay ID 001141) and 2.0 µL of cDNA sample, in total volume of 10 µL. According to previous studies assessing miRNA expression in plasma samples, U6 snRNA and miR-1228-3p were tested as housekeeping genes (endogenous controls) [42,43]. In addition to being evaluated as a VTE-related miRNA, miR-23a-3p (recognised for its stability in the presence of haemolysis) together with miR-451a (highly expressed in erythrocytes) were employed to assess potential haemolysis. A ΔCq (hsa-miR-23a-3p–hsa-miR-451a) value ≥8 indicates a high likelihood of haemolysis [44,45,46]. Since none of the samples exhibited a ratio exceeding 7, all of them were included in the analysis. The PCR conditions were as follows: 2 min at 50 °C, 10 min at 95 °C, 45 cycles of 15 s at 95 °C and 60 s at 60 °C. Measures of quality control and the methods for reliable generation of cycle threshold (Ct) values were applied as previously described [17,18].

### 2.6. Statistical Analysis

Statistical analysis and graphing were conducted using IBM SPSS Statistics for Windows (version 29, IBM Corp., Armonk, NY, USA) and GraphPad Prism (version 9.0.0, GraphPad Software Inc., La Jolla, CA, USA), respectively.

Among the tested endogenous controls, miR-1228-3p exhibited a more consistent expression, as indicated by the lowest standard deviation values. The Livak method was employed to normalise the miRNAs’ expression levels using this miRNA as an endogenous control.

Severe outliers in miRNA normalised relative expression levels were identified with the interquartile range (IQR) method and subsequently excluded. Four expression profiles were established to assess miRNA expression as a nominal variable, as previously defined [17,18].

Data normality was evaluated using the Shapiro–Wilk or Kolmogorov–Smirnov tests depending on the cohort size (N ≤ 50 and N > 50, respectively). Based on the distribution, either Spearman’s rank correlation coefficient test or Pearson’s correlation coefficient test was computed to study the relationship between the miRNAs’ expression. Results were considered relevant if *p* < 0.05 and the coefficient was ≥0.500.

VTE-free patients included those who died without experiencing CAT or remained without an event during a two-year follow-up period. Thus, live patients with less than two years of follow-up were excluded in analyses involving VTE implications. Associations of VTE occurrence and miRNAs’ expression levels with patients’ characteristics (Table 1) were evaluated using the Chi-squared test (χ^2^), excluding those who had CAT before OC diagnosis. In the analysis concerning the miRNAs, stratified evaluations according to the first treatment (surgery vs. chemotherapy) were also performed. Statistical differences in the miRNAs’ expression levels according to CAT status were analysed employing the Kruskal–Wallis test or one-way ANOVA followed by Dunnett’s test, depending on data normality. Patients who experienced a VTE event before and after being diagnosed with OC were compared, respectively, with their VTE-free counterparts. χ^2^ was employed for confirmation.

Two clinical outcome measures, namely progression-free survival (PFS) and overall survival (OS), were utilised in the study. These measures were previously defined [17,18]. The impact of VTE occurrence and miRNA expression on PFS and OS was assessed using the Kaplan–Meier method and log-rank test. Regarding the implications of miRNA expression, the most appropriate profile was appointed for each miRNA after an initial assessment of the survival curves. Additionally, the influence of miRNA expression on the risk of OC progression and mortality was evaluated using the Cox model. Multivariate Cox analyses were conducted for all the miRNAs, adjusting for the most relevant patients’ characteristics. These were identified by applying the backward Wald method. Only factors with prognostic value according to univariate Cox analyses were included in this analysis. Patients with VTE before their OC diagnosis were excluded from these analyses. Harrell’s concordance (C)-index was used to compare the predictive ability of the proposed models, with a value greater than 0.5 indicating a good predictive ability.

In the study, a significance level of 5% was set. Additionally, *p*-values falling within the range of 0.05 to 0.06 were considered marginally significant.

### 2.7. In Silico Analysis

*In silico* analyses were conducted to explore the biological implications of the relevant miRNAs. The miRTargetLink 2.0 database and the STRINGapp Protein Query from Cytoscape 3.10.2 were employed to identify miRNA targets and generate a protein–protein interaction (PPI) network for each miRNA, respectively [42]. Markov clustering (MCL) was applied to cluster proteins based on their STRING interaction scores. Subsequently, a functional enrichment analysis was performed, eliminating redundant terms with a cut-off of 0.5. The top 20 enriched terms with a false discovery rate (FDR) < 0.05 for Gene Ontology (GO) categories, Diseases, KEGG and Reactome pathways were provided for each relevant miRNA.

## 3. Results

### 3.1. Impact of VTE on Patients’ Prognosis

**Figure 1:**
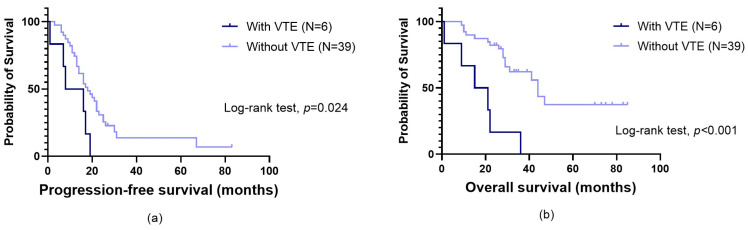
Progression-free survival (PFS) (**a**) and overall survival (OS) (**b**) by Kaplan–Meier and log-rank test among ovarian cancer (OC) patients, according to venous thromboembolism (VTE) occurrence after tumour diagnosis. (**a**) Patients with VTE had a lower PFS than their counterparts (mean PFS of 11.3 ± 2.9 months and 24.9 ± 3.8 months, respectively; log-rank test, *p* = 0.024). (**b**) Likewise, they also exhibited a lower OS (mean OS of 17.3 ± 4.9 months and 50.8 ± 5.6 months, respectively; log-rank test, *p* < 0.001). The VTE patients were treated with low-molecular-weight heparin (LMWH), except for one patient who received direct oral anticoagulants (DOACs).

Among those with a two-year follow-up (N = 48), nine (18.8%) had OC-related VTE, including seven DVT and two PE, with the majority being symptomatic (N = 6 (66.7%)). Six VTE patients (66.7%) were treated with low-molecular-weight heparin (LMWH), two (22.2%) with direct oral anticoagulants (DOACs) and one (11.1%) with vitamin K antagonists (VKAs). Those with thrombotic events previous to cancer diagnosis (N = 3) exhibited a median period between VTE and OC diagnosis of 2.0 months (minimum = 0; maximum = 5 months). Regarding patients with VTE after OC detection (N = 6), the median time to thrombogenesis was 4.5 months (minimum = 1; maximum = 24 months). Notably, except for one patient treated with DOAC, those in this latter subgroup were treated with LMWH. Thrombotic events did not impact patients’ PFS (log-rank test, *p* = 0.113), although they significantly influenced patients’ survival (log-rank test, *p* < 0.001). Specifically, those with VTE had lower OS compared to those unaffected (mean OS of 22.3 ± 4.4 months and 50.8 ± 5.6 months, respectively). Upon excluding patients with VTE before OC diagnosis, a notable association between thrombotic events and PFS emerged (log-rank test, *p* = 0.024; Figure 1a). In the same subgroup, the negative influence of VTE on OS was sustained (log-rank test, *p* < 0.001; Figure 1b). No patients’ characteristics (Table 1) were found to be predictors of OC-related VTE (χ^2^, *p* > 0.05).

### 3.2. Correlation between Baseline miRNA Expression

**Figure 2:**
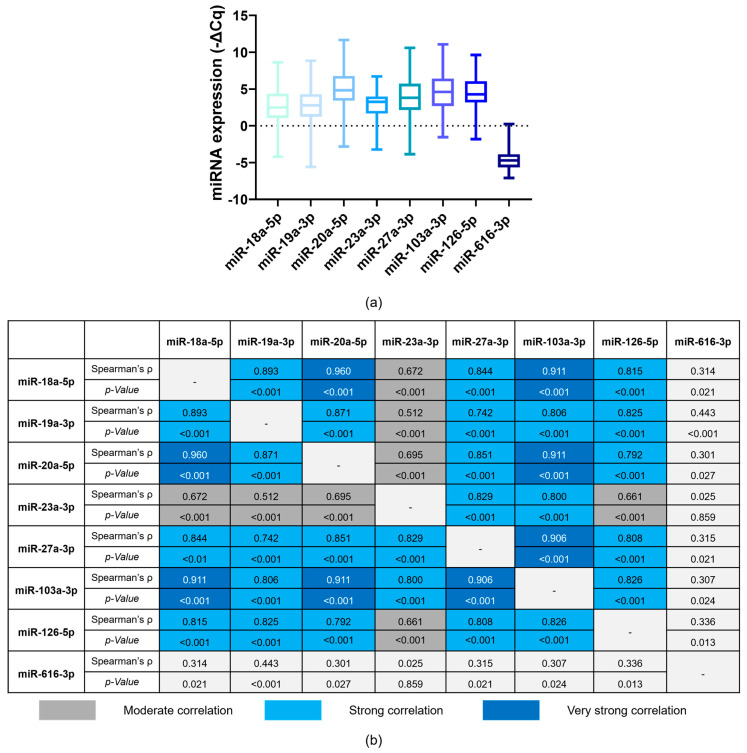
Normalised relative expression of plasma miRNAs (−ΔCq) in ovarian cancer (OC) patients preceding first-line chemotherapy (**a**) and the correlation between the baseline expression levels of these miRNAs according to Spearman’s rank correlation coefficient test (**b**). The strength of the correlations (Spearman’s *ρ* ≥ 0.500 and *p* < 0.05) was classified as follows: moderate (Spearman’s *ρ* < 0.700), strong (0.700 ≤ Spearman’s *ρ* < 0.900), and very strong (Spearman’s *ρ* ≥ 0.900).

Except for miR-616-3p, miRNAs’ expression levels were positively correlated (Spearman’s *ρ* ≥ 0.50 and *p* < 0.05; Figure 2).

### 3.3. Baseline miRNA Levels and Patients’ Characteristics

Concerning miR-18a-5p, patients with an Eastern Cooperative Oncology Group Performance Status (ECOG PS) ≤ 1 tended to exhibit high miRNA levels more frequently (profile 2, χ^2^, *p* = 0.047; profile 3, χ^2^, *p* = 0.031). Patients with lower cancer antigen 125 (CA-125) baseline levels (<913.0 U/mL)) often showed intermediate-to-high miRNA levels (profile 2, χ^2^, *p* = 0.055). Regarding miR-20a-5p, those with an ECOG PS ≤ 1 commonly displayed high miRNA levels (profile 2, χ^2^, *p* = 0.047; profile 3, χ^2^, *p* = 0.031). Patients at the International Federation of Gynecology and Obstetrics (FIGO) III/IV stages more frequently exhibited elevated miR-23a-3p levels (profile 2, χ^2^, *p* = 0.024; profile 4, χ^2^, *p* = 0.022). The same trend was noted for individuals with lower CA-125 baseline levels (profile 1, χ^2^, *p* = 0.035; profile 2, χ^2^, *p* = 0.042; profile 3, χ^2^, *p* = 0.031). Concerning miR-27a-3p, patients at III/IV stages also commonly showed high levels of this miRNA (profile 2, χ^2^, *p* = 0.028; profile 4, χ^2^, *p* = 0.022). Elevated miR-103a-3p levels were predominantly observed among patients with lower CA-125 baseline levels (profile 2, χ^2^, *p* = 0.034; profile 4, χ^2^, *p* = 0.023). High miR-616-3p levels were more frequently found among those with Khorana score (KS) ≥ 2 (profile 3, χ^2^, *p* = 0.044). As for miR-19a-3p and miR-126-5p, no significant association with patients’ characteristics was detected. It is worth noting that the first treatment (surgery vs. chemotherapy) had no significant impact on the baseline miRNA levels.

### 3.4. Baseline miRNA Levels and OC-Related VTE Susceptibility

**Figure 3:**
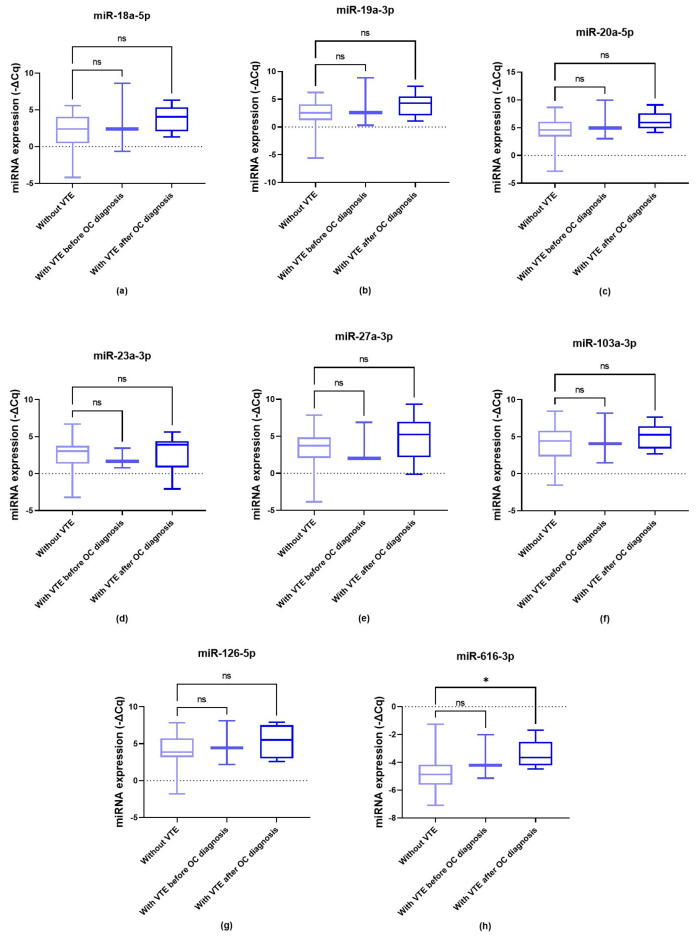
Normalised relative expression of plasma miRNAs (−ΔCq) in ovarian cancer (OC) patients preceding first-line chemotherapy, according to venous thromboembolism (VTE) status. (**a**) miR-18a-5p; (**b**) miR-19a-3p; (**c**) miR-20a-5p; (**d**) miR-23a-3p; (**e**) miR-27a-3p; (**f**) miR-103a-3p; (**g**) miR-126-5p; and (**h**) miR-616-3p; Kruskal–Wallis test, * *p* < 0.05; ns, non-significant.

Except for miR-616-3p, there were no associations between the miRNAs and VTE development according to the Kruskal–Wallis test (Figure 3a–g, *p* > 0.05). As for miR-616-3p, significantly elevated levels were observed among those who would later develop VTE compared to VTE-free patients (Kruskal–Wallis test, *p* = 0.022; Figure 3h). Regarding the analysis with χ^2^, the predictive value of miR-616-3p was confirmed (profile 1, *p* = 0.048; profile 2, *p* = 0.017; profile 3, *p* = 0.029). Furthermore, miR-20a-5p levels were significantly elevated in patients with VTE after OC diagnosis compared to VTE-free subjects (profile 2, χ^2^, *p* = 0.034). Considering the expression profile 4 of this miRNA, the likelihood test indicated a *p*-value of 0.021, reflecting a potentially significant finding (χ^2^, *p* < 0.05) if the cohort size was larger.

### 3.5. Impact of Baseline miRNA Levels on Patients’ Prognosis

**Figure 4:**
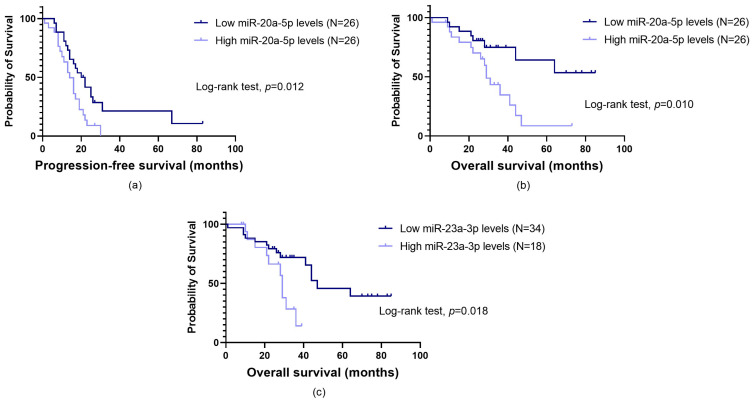
Progression-free survival (PFS) (**a**) and overall survival (OS) (**b**,**c**) by Kaplan–Meier and log-rank test in a cohort of ovarian cancer (OC) patients, according to the baseline expression levels of plasma miRNAs. (**a**) Patients with high miR-20a-5p levels had a lower PFS than their counterparts (mean PFS of 14.8 ± 1.5 months and 29.8 ± 5.4 months, respectively, log-rank test, *p* = 0.012). (**b**) These patients also had a lower OS (mean OS of 32.5 ± 4.1 months and 61.6 ± 6.7 months, respectively, log-rank test, *p* = 0.010). (**c**) Likewise, those with elevated miR-23a-3p levels had a lower survival time (mean OS of 27.1 ± 2.4 months and 53.7 ± 5.9 months, respectively, log-rank test, *p* = 0.018).

A significant association between miR-20a-5p expression and PFS was observed. Namely, patients with high miRNA levels had a lower time to disease progression than their counterparts (profile 1, log-rank test, *p* = 0.012; Figure 4a). This result was confirmed with univariate Cox regression analysis (profile 1, hazard ratio (HR) = 2.17, 95% confidence interval (CI), 1.15–4.12, *p* = 0.017). As for OS, significant associations were detected for miR-20a-5p and miR-23a-3p. Those with high miR-20a-5p levels had a lower survival time (profile 1, log-rank test, *p* = 0.010; Figure 4b). These patients presented almost a three-fold increase in the risk of death (profile 1, HR = 2.92, 95%CI, 1.23–6.93, *p* = 0.015). Likewise, elevated miR-23a-3p levels were associated with a decreased OS (profile 3, log-rank test, *p* = 0.018; Figure 4c), which was confirmed by Cox regression analysis (profile 3, HR = 2.83, 95%CI, 1.14–7.00, *p* = 0.025).

Stratified analysis based on the patient’s baseline characteristics—age (≥64 vs. <64 years), ECOG PS (>1 vs. ≤1), FIGO stage (III/IV vs. I/II), CA-125 levels (≥913.0 vs. <913.0 U/mL) and KS (≥2 vs. <2)—were conducted. All the miRNAs had a detrimental impact on PFS and/or OS when accessed at a specific subgroup (only the subgroups with significant results are shown). Among patients at early stages, a lower PFS was observed for those with high levels of miR-18a-5p (profile 3), miR-20a-5p (profile 1), miR-23a-3p (profile 3) and miR-27a-3p (profile 3) (log-rank test, *p* = 0.010, *p* = 0.010, *p* < 0.001 and *p* < 0.001, respectively). In the subgroup with lower CA-125 levels, a shorter PFS was experienced by those with high levels of miR-20a-5p (profile 1), miR-23a-3p (profile 4) and miR-27a-3p (profile 4) (log-rank test, *p* = 0.034, *p* = 0.017 and *p* = 0.028, respectively). The same was observed for those with high miR-23a-3p levels in the KS ≥ 2 subgroup (profile 4, log-rank test, *p* = 0.012).

Among the younger patients, a lower OS was observed for those with elevated levels of miR-18a-5p (profile 3), miR-19a-3p (profile 3), miR-20a-5p (profile 1), miR-23a-3p (profile 3), miR-27a-3p (profile 3), miR-103a-3p (profile 3), miR-126-5p (profile 3) and miR-616-3p (profile 3) (log-rank test, *p* = 0.001, *p* < 0.001, *p* = 0.020, *p* = 0.002, *p* = 0.019, *p* < 0.001, *p* = 0.001 and *p* = 0.011, respectively). In the subgroup with ECOG PS ≤ 1, OS was lower in patients with high levels of miR-20a-5p (profile 1) and miR-23a-3p (profile 3) (log-rank test, *p* = 0.031 and *p* = 0.036, respectively). Among patients at early cancer stages, those with high levels of miR-18a-5p (profile 3), miR-19a-3p (profile 3), miR-20a-5p (profile 1), miR-23a-3p (profile 4) and miR-27a-3p (profile 4) exhibited a lower survival time (log-rank test, *p* = 0.011, *p* = 0.045, *p* = 0.011, *p* = 0.045 and *p* = 0.045, respectively). In the subgroup with lower CA-125 levels, patients with high levels of miR-20a-5p had a lower OS (profile 1, log-rank test, *p* = 0.031). The same was seen in the KS < 2 subgroup for those with elevated levels of miR-20a-5p (profile 1) and miR-616-3p (profile 3) (log-rank test, *p* = 0.030 and *p* = 0.008, respectively).

Multivariate Cox analyses were performed for all the miRNAs, adjusting for relevant demographic and clinicopathological factors. Significant predictors of disease progression included patients’ age (≥64 years, HR = 1.98, 95%CI, 1.05–3.76, *p* = 0.036), FIGO stage (III/IV, HR = 2.71, 95%CI, 1.12–6.60, *p* = 0.028), first treatment (surgery, HR = 0.33, 95%CI, 0.17–0.64, *p* = 0.001) and surgery (yes, HR = 0.13, 95%CI, 0.06–0.30, *p* < 0.001). In a multivariate Cox analysis applying the backward Wald method, surgery (yes vs. no) was the most relevant predictor of disease progression (clinic model A1). This variable was defined as having or not having the intervention during the patient’s first-line treatment. Regarding the risk of death, patients’ age (≥64 years, HR = 6.12, 95%CI, 2.20–16.98, *p* < 0.001), hormonal status (post-menopause, HR = 5.66, 95%CI, 1.31–24.47, *p* = 0.020), surgery (yes, HR = 0.14, 95%CI, 0.05–0.37, *p* < 0.001) and platinum sensitivity (no, HR = 5.49, 95%CI, 2.68–11.24, *p* < 0.001) had a significant predictive value. Among these factors, patients’ age, surgery and platinum sensitivity were the most relevant predictors of mortality (clinic model B1). Furthermore, by focusing on factors with available data at the start of the patient’s treatment (i.e., dismissing surgery and platinum sensitivity), primary treatment (clinic model A2) and patients’ age (clinic model B2) emerged as the most informative factors concerning the risk of disease progression and death, respectively. Considering these models, multivariate Cox analyses, including the miRNAs (integrative models), were conducted (Table 3). The C-index was computed to compare the predictive ability of the models for disease progression and death. The integrative models (including data on the baseline miRNA levels) outperformed their clinical counterparts in predictive capacity. Integrative model A1, the top predictor for disease progression, exhibited a 5% improvement in predictive ability over its clinical counterpart (c-index, 0.952 vs. 0.902; Table 3). Notably, integrative model A2 demonstrated a 34% increase in predicting disease progression risk compared to its clinical counterpart (c-index, 0.898 vs. 0.671; Table 3). For predicting the risk of death, the best model—integrative model B1—showed a 3% enhancement (c-index, 0.848 vs. 0.821; Table 3).

### 3.6. In Silico Analysis

**Figure 5:**
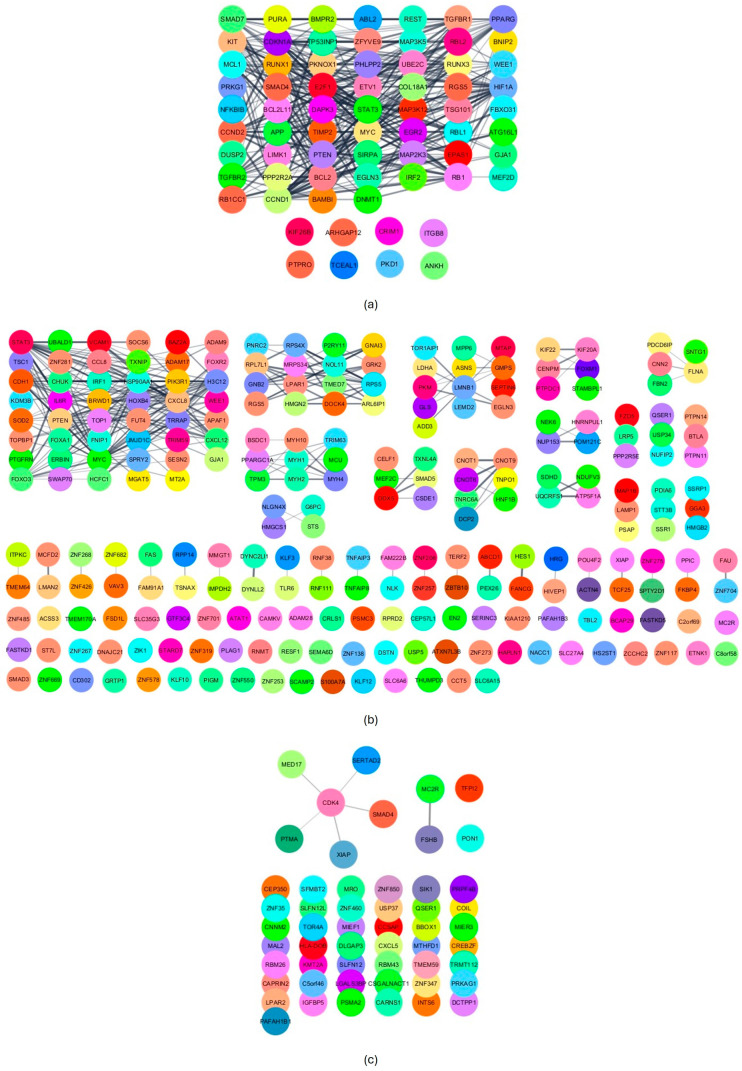
Protein–protein interaction (PPI) networks of miR-20a-5p (**a**), miR-23a-3p (**b**) and miR-616-3p (**c**) validated targets.

**Figure 6:**
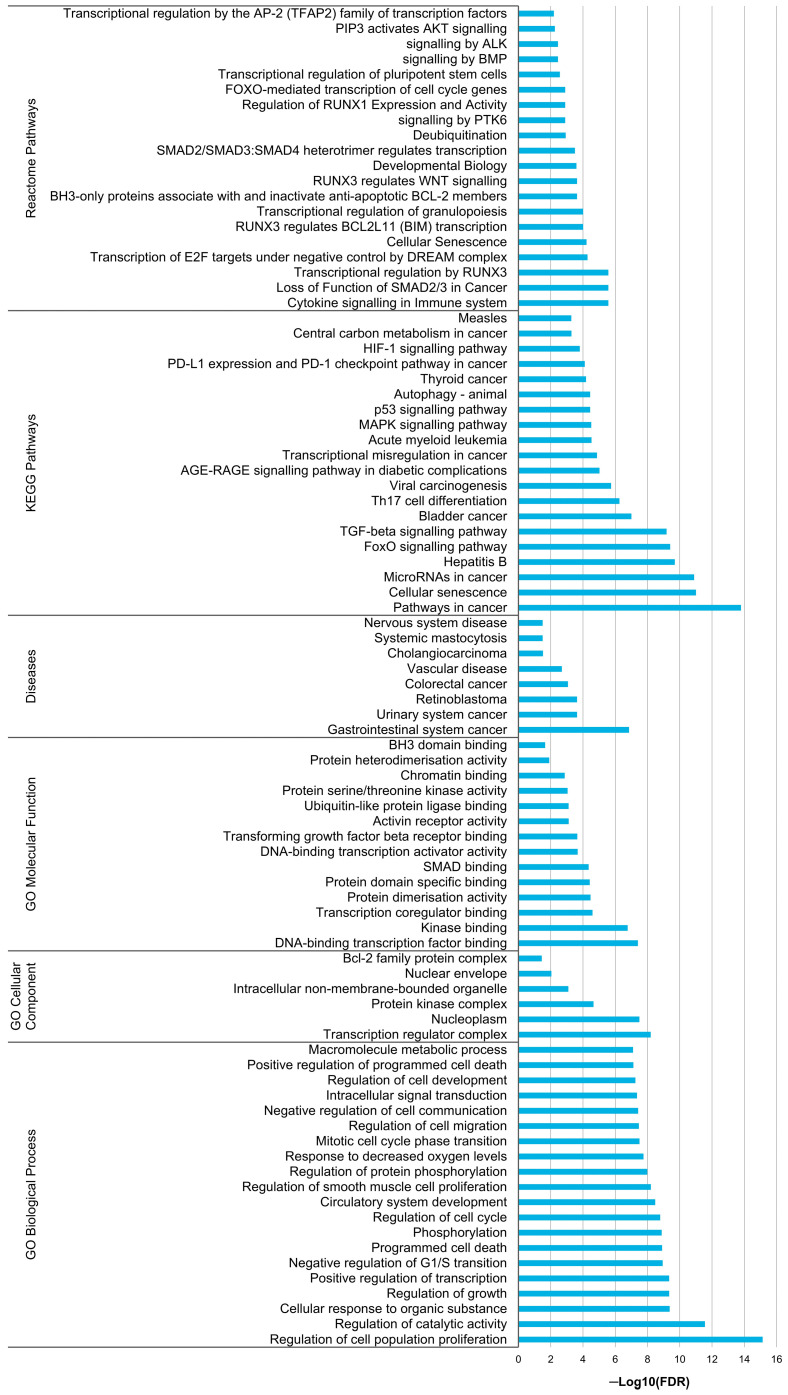
Functional enrichment analysis for miR-20a-5p.

**Figure 7:**
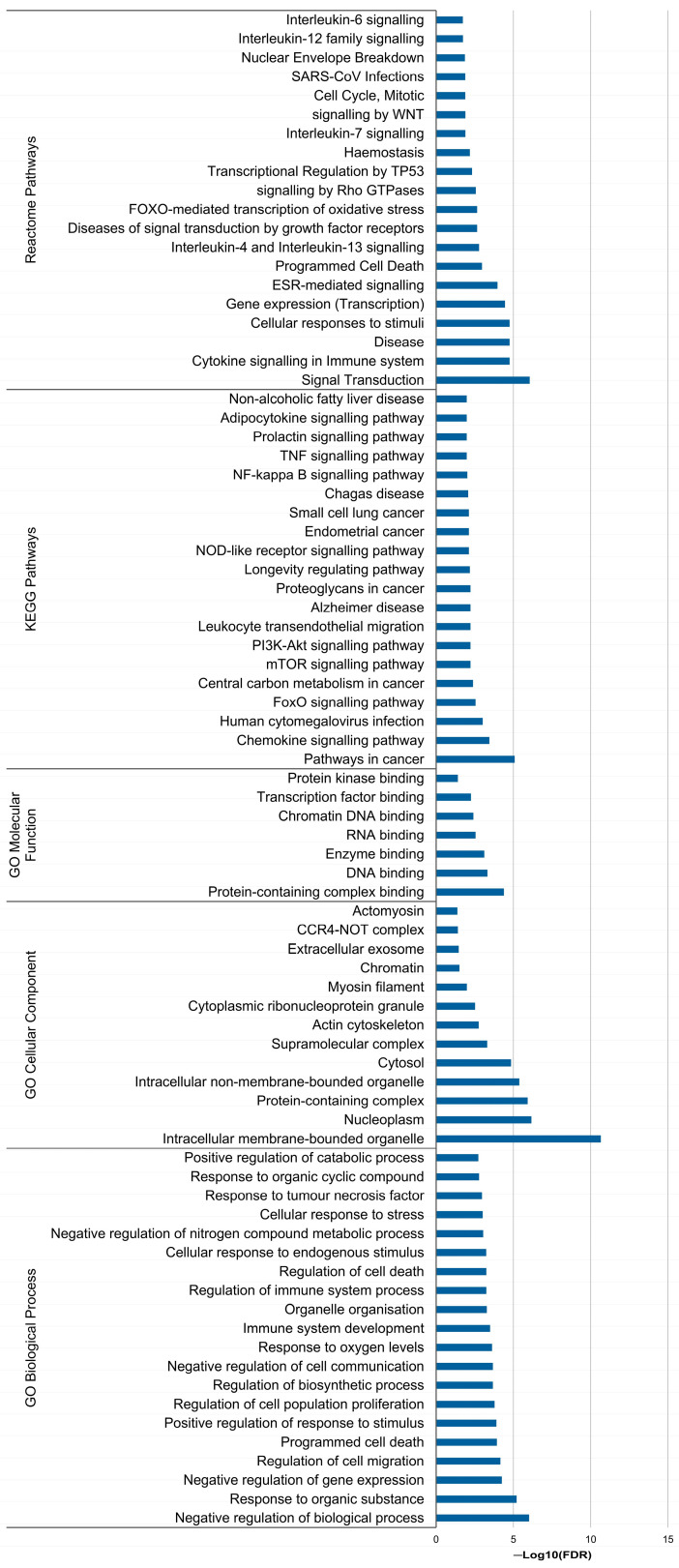
Functional enrichment analysis for miR-23a-3p.

In this study, miR-20a-5p, miR-23a-3p and miR-616-3p exhibited a more predominant role. Thus, *in silico* analyses were conducted to explore their potential biological implications. For miR-20a-5p, since it had more than 300 validated targets (cut-off defined to ensure clear visualisation of the targets), only the strongly validated ones were considered (N = 68). For miR-23a-3p and miR-616-3p, 248 and 53 targets (including TFPI2 as a strongly validated target of miR-616-3p) were reported, respectively. The PPI networks generated for the targets of miR-20a-5p (68 nodes and 317 edges, *p* = 1.0 × 10^−16^; Figure 5a) and miR-23a-3p (246 nodes and 312 edges, *p* = 7.9 × 10^−7^; Figure 5b) had significant enrichment. The functional enrichment analysis focused on the clusters (60 nodes and 317 edges for miR-20a-5p and 183 nodes and 312 edges for miR-23a-3p) identified 250 and 154 enriched terms for miR-20a-5p and miR-23a-3p, respectively. The top 20 enriched terms for each miRNA concerning GO categories, Diseases, KEGG and Reactome pathways are presented in Figure 6 and Figure 7. Notably, no enriched term regarding Diseases was found for miR-23a-3p (Figure 7). Moreover, no significant enrichment was found for miR-616-3p due to the limited interaction among its targets (52 nodes and six edges, *p* = 0.74; Figure 5c), which prevented further functional enrichment analysis.

## 4. Discussion

Cancer is linked to a broad range of haemostatic issues, with the tumour coagulome fuelling tumourigenesis. Recent investigation delves into understanding how tumour cells “educate” the haemostatic system to facilitate cancer progression [47]. This study examined the implications of VTE and the role of miRNAs targeting the TF pathway in OC patients. A CAT incidence of nearly 19% was observed, which is consistent with the literature reporting an incidence range of 10% to 30% [3]. Particularly among those with VTE after OC diagnosis, VTE negatively affected their PFS and OS (long-rank test, *p* = 0.024 and *p* < 0.001, respectively). Unlike survival time, thrombotic events did not significantly impact patients’ PFS when considering patients with VTE before OC diagnosis (log-rank test, *p* = 0.113). The use of anticoagulants prior to treatment may have potential effects, yet further research is needed to elucidate the underlying biological mechanisms. Notably, among those with VTE after OC diagnosis, LMWH was the commonly chosen anticoagulant therapy, aligning with the existing literature. Although with some data inconsistency, LMWH is thought to be less effective than DOACs among cancer patients, while the latter is seemingly linked to a heightened risk of bleeding and additional complications [48,49]. Regarding CAT prediction, neither demographic and clinicopathological factors nor KS were reliable predictors of OC-related VTE in this study, an observation that warrants investigation in larger cohorts.

Except for miR-616-3p, some correlation was observed among the evaluated plasma miRNAs. MiR-18a-5p, miR-19a-3p and miR-20a-5p exhibited strong correlations. Consistently, they belong to the miR-17-92 cluster (chromosome 13q31.3) and are experimentally validated to target *F3* [50]. In addition, miR-19a-3p has been associated with CAT development [28]. Regarding miR-27a-3p, this *F3-* and *TFPI1*-targeting miRNA is a potential diagnostic biomarker of VTE in the general population [33,34,35]. Concerning miR-103a-3p, its circulating levels have been implicated in first and recurrent VTE events, as well as CAT [28,36,37]. This miRNA potentially modulates the risk of thrombosis by influencing endothelial cell function and targeting genes regulating TF expression [51,52]. Also, according to miRmap, miRwalk and DIANA-TarBase, miR-103a-3p targets *TFPI2* expression. As for miR-126-5p, increased circulating levels of this *F3*-targeting miRNA have been found to be a diagnostic and prognostic biomarker of VTE in the general population, as well as a predictive biomarker of CAT [22,25,32,38,39]. Overall, miR-18a-5p, miR-19a-3p, miR-20a-5p, miR-27a-3p, miR-103a-3p and miR-126-5p seem to all impact *F3* expression, which may partially explain the correlation between these miRNAs. MiR-23a-3p and miR-616-3p are validated *TFPI2*-targeting miRNAs [31,40,41]. The former showed a moderate correlation with miR-18a-5p, miR-19a-3p, miR-20a-5p and miR-126-5p and a strong correlation with miR-27a-3p (both are members of the miR-23a∼27a∼24-2 cluster, chromosome 9q22) and miR-103a-3p, whereas the latter did not correlate with any of the miRNAs [53].

In the study, miR-20a-5p, miR-23a-3p and miR-616-3p were the most relevant miRNAs when considering the entire cohort. Starting with miR-20a-5p, this validated *F3*-targeting miRNA is reported to inhibit and promote OC cell proliferation, migration, invasion and cisplatin resistance, depending on the context [54,55,56]. Xie et al. (2014) [57] have found that elevated circulating miR-20a levels were linked to poor OS. In the present study, high plasmatic levels of miR-20a-5p were observed among patients with an ECOG PS ≤ 1 (profile 2, χ^2^, *p* = 0.047; profile 3, χ^2^, *p* = 0.031). This upregulation was significantly associated with VTE development (profile 2, χ^2^, *p* = 0.034), which could be partially explained by *F3* and *TFPI2* targeting, the latter with less evidence. In addition to TF, recent studies have highlighted TFPI2 as a predictive and diagnostic biomarker of OC-related VTE, particularly when combined with D-dimer levels, as well as a diagnostic and prognostic marker of OC [58,59,60]. Notably, most data link this haemostatic protein to the pathogenesis of ovarian clear cell carcinoma—the OC subtype most strongly associated with VTE. In this subtype, TFPI2 is overexpressed [61,62,63].

In this study, miR-20a-5p upregulation was also associated with a faster disease progression (profile 1, log-rank test, *p* = 0.012) and a decreased survival time (profile 1, log-rank test, *p* = 0.010), which is in line with the findings of Xie et al. (2014) [57]. Patients with heightened levels faced a two-fold increase in the risk of OC progression (profile 1, *p* = 0.017). Based on the stratified analysis, the miRNA appears to have a more preponderant influence on disease progression among individuals at early cancer stages and with lower CA-125 levels. Those with elevated levels of miR-20a-5p also experienced nearly a three-fold rise in the risk of mortality (profile 1, *p* = 0.015). This adverse effect was more pronounced in younger patients, those at early cancer stages, with an ECOG PS ≤ 1, lower CA-125 levels and a KS < 2. Interestingly, Xie et al. (2014) [57] found that elevated serum miR-20a expression was significant only in OC patients with CA-125 levels ≤ 500 U/mL. Overall, this miRNA appears to be more significant in the pre-metastatic phase and/or when the tumour burden is low. This supports our previous findings that certain VTE-related biomarkers may have a greater impact before metastasis, potentially aiding in the spread of tumour cells [64,65]. As for patients’ age and KS, the former is a significant factor influencing the risk of thrombogenesis, whereas KS stands out as the most extensively studied risk assessment model (RAM) for CAT, which corroborates the influence of haemostatic mechanisms in miR-20a-5p function [4]. This study’s best predictive model for disease progression—integrative model A1—combined information on surgery (yes vs. no) and baseline levels of miR-19a-3p and miR-20a-5p. Likewise, the integrative model A2, designed for use at the beginning of first-line treatment, showed a 34% improvement in predictive accuracy over its clinical counterpart. This model encompasses data on primary treatment options (surgery vs. chemotherapy) and baseline miR-20a-5p levels, highlighting the potential clinical significance of this miRNA for an early prognosis evaluation of OC patients.

According to the *in silico* analysis, miR-20a-5p seems to be implicated in cell proliferation, differentiation, migration and senescence/apoptosis, also participating in host immune response. Importantly, this miRNA appears to regulate cell cycle and transcription activity and play an important role in circulatory system development. Overall, miR-20a-5p seems to be involved in several signalling pathways that foster both tumour progression and cancer-associated hypercoagulation, particularly HIF-α, PI3K/Akt and TGF-β/Smad pathways [66,67,68,69]. All in all, focusing on the TF pathway, the deregulation of TF and TFPI2, both targeted by miR-20a-5p, might contribute to the tumour coagulome and OC progression. According to the literature, cells actively or passively release miRNAs into the extracellular space through four main mechanisms: miRNAs encapsulated within extracellular vesicles, complexed with the Argonaute2 protein (Ago2), bound to high-density lipoprotein (HDL) or associated with the RNA-binding protein nucleophosmin (NPM1). Passive release occurs due to tissue injury, chronic inflammation, apoptosis or necrosis, which are common in cancer [70]. The requirement to maintain TF expression, combined with the upregulated plasma levels of miR-20a-5p linked to a poor prognosis, may suggest an active release of this miRNA from the OC cells, key haemostatic and/or immune players into the bloodstream, a hypothesis that requires further investigation. Currently, the expression pattern of *TFPI2* in the more aggressive ovarian tumours is unclear when considering all histological subtypes. A better understanding of the specific molecular and cellular environment in which miR-20a-5p operates, along with its expression dynamics, is crucial to effectively target it for prognostic and therapeutic purposes.

Like miR-20a-5p, miR-23a-3p is reported to have contradictory roles in OC. Overexpression of this validated *TFPI2*-targeting miRNA in OC tissue has been associated with tumour cell proliferation, invasion, migration and chemoresistance, as well as a poor PFS [71,72,73,74,75]. However, pro-apoptotic and chemosensitive effects have also been observed in certain contexts within OC cells [76]. Regarding the impact of circulating miR-23a-3p in OC patients, data are limited. This study found that elevated miRNA levels were more common among patients at advanced cancer stages and those with lower CA-125 levels, an intriguing and inconclusive finding. A previous study reported that miR-23a downregulates *TFPI2* expression in pancreatic cancer cells and exacerbates their malignant characteristics, thus acting as an oncogene [31]. Furthermore, elevated plasma levels of miR-23a-3p have been shown to predict the occurrence of CAT [32]. In this study, no impact on venous thrombogenesis was observed. Nevertheless, upregulated miR-23a-3p levels were associated with a decreased survival time (profile 3, log-rank test, *p* = 0.018). Patients with high levels face nearly a three-fold increase in the risk of death (profile 3, *p* = 0.025). According to stratified analysis, the miRNA appears to influence disease progression among younger patients, those with lower CA-125 levels and with KS ≥ 2. Furthermore, elevated levels of the miRNA seem to negatively impact the survival time of younger patients with an ECOG PS ≤ 1 and at early cancer stages. Overall, like miR-20a-5p, miR-23a-5p may have an important role in a pre-metastatic setting. Lastly, the multivariate Cox analysis indicated that miR-23a-3p is an independent predictor of risk of death.

According to the *in silico* analysis, miR-23a-3p is mainly involved in regulating immune response, cell proliferation, migration and apoptosis, which aligns with the existing evidence [71,72,73,74,75,76]. Interestingly, miR-23a-3p’s involvement in haemostasis was corroborated. Also, this miRNA participates in PI3K/Akt and FOXO signalling pathways, which are implicated in cancer and thrombogenesis [68,77]. All in all, miR-23a-3p appears to have a context-dependent role in OC pathways, echoing the functions of its target, TFPI2. However, further studies are necessary to elucidate miRNA expression dynamics and its mechanism of release in peripheral blood.

Recently, miR-616 has been addressed as a cancer-associated miRNA, playing an oncogenic role in gastric, ovarian and breast cancers [78,79,80]. In OC, elevated miR-616 levels correlated with poor differentiation, invasion, migration and metastasis in experimental settings [79]. However, data on circulating miR-616-3p in OC patients are scarce. In this study, high levels of this miRNA were more common among those with KS ≥ 2 (profile 3, χ^2^, *p* = 0.044), suggesting a potential role in CAT pathogenesis. Concordantly, its upregulated levels were found among patients who later developed VTE after OC diagnosis compared to VTE-free patients (Kruskal–Wallis test, *p* = 0.022; profile 1, *p* = 0.048; profile 2, *p* = 0.017; profile 3, *p* = 0.029). This prothrombotic effect could be due to *TFPI2*, one of the few strongly validated targets of miR-616-3p [41]. Similarly, a prior study conducted by our research group found that elevated pre-chemotherapy levels of MEG8 (a long non-coding RNA targeting *TFPI2* expression) in peripheral blood cells (PBCs) were predictive of VTE after an OC diagnosis [17,81].

While no significant association was found in the overall cohort regarding its influence on patients’ prognosis, increased levels of miR-616-3p were associated with a lower OS in younger patients and those with KS < 2 (long-rank test, *p* < 0.05). Consistently, the best predictive model for the risk of death integrated information on patients’ age, surgery, platinum sensitivity and baseline miR-616-3p levels. Although it only yielded a marginal 3% enhancement in predictive accuracy compared to the clinical model alone, the integrated model B1 demonstrated the independent predictive capability of this miRNA. Unfortunately, *in silico* analysis was unfeasible due to the limited interactions among the miRNA targets. Overall, miR-616-3p could be a valuable tool for predicting OC-related VTE and improving prognostic accuracy. More investigation is needed to elucidate the miRNA expression dynamics and its role in OC.

According to the stratified analyses, miR-18a-5p, miR-19a-3p, miR-27a-3p, miR-103a-3p and miR-126-5p appear to be particularly relevant in the pre-metastatic phase and among younger patients, consistent with findings for miR-20a-5p, miR-23a-3p and miR-616-3p. Furthermore, miR-19a-3p was a predictor of the risk of OC progression in the multivariate Cox analysis adjusting for surgery (yes vs. no) and baseline miR-20a-5p (integrative model A1). Whether these results indicate a context-dependent role or are influenced by the correlations between the miRNAs or the cohort size remains to be determined.

This study presented some limitations. Due to the relatively low incidence of OC and the need to control for major confounders associated with CAT development, the cohort size was small, impacting the statistical power. This study’s retrospective nature prevented the evaluation of miRNA expression levels near the occurrence of VTE, which would offer valuable insights into their diagnostic and prognostic potential concerning thrombosis. Also, no active VTE screening was conducted, potentially underestimating asymptomatic events. Moreover, *in silico* analyses have intrinsic limitations. Given the complexity of OC and CAT pathogenesis, other genes and pathways with an impacting role may have been overlooked. As previously mentioned, a single miRNA can target several mRNAs, influencing multiple biological pathways. More real-world data are needed for an integrative view of the related mechanisms. Despite these constraints, the study has notable strengths in accounting for major risk factors associated with VTE and CAT.

## 5. Conclusions

Among gynaecological tumours, OC is the most lethal. Investigating haemostasis deregulation in cancer progression is promising for identifying OC biomarkers and therapeutic targets. Meanwhile, circulating miRNAs have emerged as valuable tools for understanding not only CAT pathogenesis but also ovarian tumourigenesis. These RNAs are accessible, resistant to environmental conditions and allow for minimally invasive sample collection, making them suitable for routine clinical use and longitudinal monitoring. This study evaluated plasma expression levels of miRNAs targeting the TF coagulation pathway in OC patients. The findings confirmed that VTE negatively impacts their prognosis, affecting both disease progression and survival. Baseline levels of miR-20a-5p and miR-616-3p were shown to predict OC-related VTE, with miR-20a-5p being a relevant indicator of OC progression and miR-616-3p significantly impacting survival, which underscores the bidirectional relationship between thrombosis and ovarian tumourigenesis. Additionally, miR-23a-3p influenced patients’ prognosis, though it did not affect thrombogenesis. Although more studies with larger cohorts are required to validate their clinical applicability, these miRNAs appear to be valuable for tailoring thromboprophylaxis and could potentially aid in the selection of the most effective anticoagulation therapy to prevent recurrent events. Importantly, they could also enhance prognosis accuracy and potentially serve as therapeutic targets for OC management. Overall, this could pave the way for more personalised medicine. To our knowledge, this is the first study to address the implications of plasma miRNAs targeting the TF coagulation pathway in OC patients, with the findings suggesting a pivotal role of TF and TFPI2 in OC pathogenesis.

## Figures and Tables

**Table 1:** Demographic and clinicopathological data of OC patients (N = 55).

Variable	N (%)
**Age at OC diagnosis (years)** †	63.8 ± 11.9
≥64	31 (56.4)
**Hormonal status at OC diagnosis**	
Post-menopausal	43 (78.2)
**Baseline BMI (kg/m^2^)** †	26.6 ± 4.7
≥27.0	22 (40.0)
**ECOG PS at OC diagnosis**	
>1	7 (12.7)
**OC-related germline mutations**	6 (10.9)
**Histology**	
Serous	46 (83.6)
Clear cell	3 (5.5)
Endometroid	2 (3.6)
Mixed	2 (3.6)
Unusual	2 (3.6)
**Histological grade**	
High	51 (92.7)
**FIGO stage** §	
I/II	11 (20.0)
III/IV	44 (80.0)
**Baseline CA-125 levels (U/mL)** ‡	
≥913.0	27 (49.1)
**Baseline aPTT (s)** ‡	
≥27.2	24 (43.6)
**Baseline PT (s)** ‡	
≥14.2	22 (40.0)
**Baseline INR** ‡	
≥1.06	23 (41.8)
**KS** ‡‡	
≥2	21 (38.2)
**Anticoagulation therapy** ††	3 (5.5)
**Platelet anti-aggregation therapy**	7 (12.7)
**First-line treatment**	
Surgery and adjuvant chemotherapy	24 (43.6)
Neoadjuvant chemotherapy, surgery and adjuvant chemotherapy	15 (27.3)
Chemotherapy only	12 (21.8)
Neoadjuvant chemotherapy and surgery	4 (7.3)
**Platinum sensitivity** §§	39 (70.9)
**Maintenance therapy**	
PARP inhibitors	17 (30.9)
bevacizumab	9 (16.4)

† Given the normal distribution, the categories were defined based on the mean value. Age at OC diagnosis and baseline BMI were also presented as mean ± standard deviation. § Based on FIGO Cancer Report 2021 [19]. ‡ Since the variables were non-normally distributed, the categories were defined based on the median value. §§ Defined as OC progression after six months following the completion of first-line platinum-based chemotherapy [3]. ‡‡ Determined for patients with available data using these parameters: the cancer site, baseline haemoglobin levels, BMI and platelet and leukocyte count. The cut-off of two points was considered [20]. †† Patients with cancer-associated thrombosis before OC diagnosis. Certain patients lacked specific information: eleven had missing data for aPTT, ten for PT and INR, five for platinum sensitivity, two for OC-related inherited mutations and histological grade, and one for BMI and CA-125 levels. Abbreviations: aPTT, activated partial thromboplastin; BMI, body mass index; CA-125, cancer antigen 125; ECOG PS; Eastern Cooperative Oncology Group Performance Status; FIGO, International Federation of Gynecology and Obstetrics; INR, international normalised ratio; KS, Khorana score; N, number of patients; OC, ovarian cancer; PT, prothrombin time.

**Table 2:** Selected miRNAs and their targets.

miRNAs	Target Gene	miRNA–mRNA Interaction on Physio-Pathological Settings	Predicted miRNA–mRNA Interactions				Experimentally Validated miRNA–mRNA Interactions *		VTE **
			TargetScanHuman	MiRDB	miRmap	miRwalk	DIANA-TarBase	miRTarBase	
			Site Type (Context++ Score Percentile)	Target Score	miRmap Score	Score (Region)	MicroT Score	Level of Evidence	
miR-18a-5p	*F3*	-	7mer-m8 (98)	51	98.28	0.92 (3′ UTR)	0.71	Less strong	-
miR-19a-3p	*F3*	[21,22,23,24,25,26,27]	8mer (98)	92	85.15	-	0.97	-	B [28]
miR-20a-5p	*F3*	[24,29,30]	8mer (99)	95	84.88	-	0.25	Less strong	-
	*TFPI2*	-	7mer-A1 (88)	-	87.88	-	0.74	-	
miR-23a-3p	*TFPI2*	[31]	7mer-m8 (92)	-	52.23	0.92 (CDS)	0.75	-	B [32]
miR-27a-3p	*F3*	-	7mer-m8 (92)	57	79.20	0.85 (3′ UTR)	0.83	-	A [33]
	*TFPI1*	[34,35]	8mer (97)	72	84.00	0.92 (3′ UTR)	0.96	-	
miR-103a-3p	*TFPI2*	-	-	-	52.23	1.00 (CDS)	0.72	-	C [28,36,37]
miR-126-5p	*F3*	[22,25]	-	-	-	-	-	-	C [32,38,39]
miR-616-3p	*TFPI2*	[40,41]	7mer-m8 (96)	-	48.40	-	-	Strong	-

* When available, the MicroT Score from DIANA-TarBase and the level of evidence from miRTarBase were provided for *F3*, *TFPI1* and *TFPI2*. ** A: VTE in the general population; B: VTE in cancer patients; C: VTE in the general population and among cancer patients. Abbreviations: CDS, coding sequence; miRNA, microRNA; mRNA, messenger RNA; UTR, untranslated region; VTE, venous thromboembolism.

**Table 3:** Multivariable Cox regression analysis on the risk of disease progression and mortality.

Model (N)	Variable	aHR	95%CI	*p*-Value	C-Index		Event
					Clinic Model	Integrative Model	
**Integrative model A1** (47)	Surgery (Yes vs. no ^1^)	0.16	0.07–0.37	<0.001	0.902	**0.952**	**Risk of** **disease** **progression**
	miR-19a-3p *** (Low vs. high levels ^1^)	4.85	1.37–17.16	0.014			
	miR-20a-5p * (High vs. low levels ^1^)	6.13	1.72–21.83	0.005			
Integrative model A2 (52)	Primary treatment (Surgery vs. chemotherapy ^1^)	0.33	0.17–0.64	0.001	0.671	0.898	
	miR-20a-5p * (High vs. low levels ^1^)	2.17	1.14–4.15	0.019			
**Integrative** **model B1** (46)	Patients’ age at OC diagnosis (≥64 vs. <64 years ^1^)	7.43	2.24–24.65	0.001	0.821	**0.848**	**Risk of** **death**
	Surgery (Yes vs. no ^1^)	0.16	0.05–0.47	<0.001			
	Platinum sensitivity (No vs. yes ^1^)	4.90	1.96–12.24	<0.001			
	miR-616-3p *** (High vs. low levels ^1^)	3.72	1.23–11.21	0.020			
Integrative model B2 (52)	Patients’ age at OC diagnosis (≥64 vs. <64 years ^1^)	6.03	2.15–16.88	<0.001	0.699	0.713	
	miR-23a-3p ** (High vs. low levels ^1^)	2.68	1.06–6.77	0.037			

^1^ Reference group. * Profile 1; ** Profile 3; *** Profile 4. Clinic models incorporated only demographic and clinicopathological factors, while integrative models also encompassed the baseline miRNAs’ expression. Bold models were deemed the most suitable. Abbreviations: aHR, adjusted hazard ratio; CI, confidence interval; N, number of patients; OC, ovarian cancer.

## Data Availability

The data presented in this study are available upon request from the corresponding author.
